# A European study on alcohol and drug use among young drivers: the TEND by Night study design and methodology

**DOI:** 10.1186/1471-2458-10-205

**Published:** 2010-04-26

**Authors:** Roberta Siliquini, Simone Chiadò Piat, Francisco Alonso, Axel Druart, Marcin Kedzia, Antonio Mollica, Valeria Siliquini, Daniel Vankov, Anita Villerusa, Lamberto Manzoli, TEND Group

**Affiliations:** 1 Department of Public Health, University of Torino, Torino, Italy; 2 INTRAS, Universitat de València-UVEG, Valencia, Spain; 3 Responsible Young Drivers, Brussels, Belgium; 4 Prezes Fundacji "Kierowca Bezpieczny", Warsaw, Poland; 5 CONSEPI S.p.A., Susa (TO), Italy; 6 S&T soc. coop., Torino, Italy; 7 Open Youth, Sofia, Bulgaria; 8 Dep. of Public health and epidemiology, Riga Stradins University, Riga, Latvia; 9 Section of Epidemiology and Public Health, University of Chieti, Chieti, Italy

## Abstract

**Background:**

Young individuals are the age group with the highest risk of car accidents. One of main explanations relies on the use of psychoactive substances (alcohol, illegal and medicinal drugs), which are known to be major risk factors of road accidents, and whose consumption is almost universally more common among younger drivers. Although the correlation between psychoactive substances use and decrease in driving performance has been established in controlled experimental or laboratory settings, few studies were conducted in naturalistic circumstances. The TEND by Night project has been designed to evaluate the relationship between driving performance and psychoactive substances assumption in young drivers enrolled at typical places of consumption.

**Methods/Design:**

The TEND by Night project, endorsed by the European Commission, is a multidisciplinary, multi-centric, cross-sectional study conducted in six European countries (Italy, Belgium/Netherlands, Bulgaria, Spain, Poland and Latvia). The study population consists of 5000 young drivers aged 16-34 years, attending recreational sites during weekend nights. The intervention is based on the portal survey technique and includes several steps at the entrance and exit of selected sites, including the administration of semi-structured questionnaires, breath alcohol test, several drug assumption test, and measurement of the reaction time using a driving simulator. The main outcome is the difference in reaction time between the entrance and exit of the recreation site, and its correlation with psychoactive substances use. As a secondary outcome it will be explored the relationship between reaction time difference and the amount of consumption of each substance. All analyses will be multivariate.

**Discussion:**

The project methodology should provide some relevant advantages over traditional survey systems. The main strengths of the study include the large and multicentric sample, the objective measurement of substance assumption (which is typically self-reported), the application of a portal survey technique and the simultaneous evaluation of several psychoactive substances.

## Background

Young individuals are the age group with the highest risk of car accidents, as documented by crash and injury statistics in all high-income countries [1-3], in which motor vehicle-related injuries represent the leading cause of death in people aged less than 30 years [4,5].

One of main explanations relies on the use of psychoactive substances (alcohol, illegal and medicinal drugs), which are known to be major risk factors of road accidents [6-12], and whose consumption is almost universally more common among younger drivers [13-16].

Although it is well known that psychoactive substances impair driving performance in controlled experimental or laboratory settings [17-21], only few studies investigated the driving performance of psychoactive substances consumers in naturalistic circumstances [22,23]. In particular, Brookhuis et al. assessed the driving performance of 17 MDMA users in an advanced driving simulator before and after visiting a rave party [22], while Trerotoli and colleagues investigated the association between alcohol consumption and level of attention in 217 young subjects after a night in discotheque [23]. These studies considered only a single substance consumption and involved relatively small samples of young participants in regional settings.

We designed an international multicentric cross-sectional survey - the TEND (Dark, Dance, Disco, Dose, Drugs, Drive, Danger, Damage, Disability, Death) by Night project - in order to evaluate the relationship between driving performance and alcohol or other psychoactive substances (cocaine, methamphetamine/MDMA, THC, amphetamine, opiates, benzodiazepines) assumption in young drivers enrolled at typical places of consumption. The secondary aim of the project is to contribute at increasing young peoples' awareness on the potential influence of alcohol and psychoactive substances on their driving capacity through the dissemination of correct and effective information.

The present paper describes the research protocol of the project, which is currently on course.

## Methods/Design

The TEND by Night project is a multidisciplinary, multicentric, cross-sectional study that is performed in six European countries - Italy, Belgium/Netherlands, Bulgaria, Poland, Spain and Latvia - the last of which, however participated in the study design but not in the intervention. The project, which is endorsed by the European Commission - Public Health Executive Agency "see Additional file 1", will collect data on young people alcohol and various drugs misuse proximal to the habitual site of consumption and it will measure the reaction time, a specific driving performance indicator, using a driving simulator. The research hypothesis is that young drivers who assume alcohol and/or drugs in recreational places show a delay in their reaction time which is proportional to the amount of consumption.

### Research universe and study sample

The study population consists of subjects aged 16 to 34 years, who own a driving license and attend recreational sites during weekend nights. The required sample size was calculated using the Schlesselmann formula [24] for known population sizes, assuming a two-sided alpha = 0.05, a statistical power = 90%, losses or missings = 25%, and taking into account the need to stratify for each country and to adjust for several potential confounders in the multivariate analysis. With these parameters, the estimated sample size was conservatively set at 1000 individuals for each of the five actively involved countries, for a total of 5000 subjects.

In each nation, a map of the recreational meeting places (representative of the main territorial contexts involved, with relation to the target population) was realized on the basis of official regional lists. After a drawing up of a first selection, the final choice was made according to the willingness of the clubs' owners.

### The TEND by Night intervention

The TEND by Night intervention [25] is based on the portal survey technique [26-28], applied in selected youth recreational places during weekend nights by a survey staff. Every intervention includes several steps at the entrance and exit of the recreational places. When people enter the club, the scheduled activities are: participants' voluntary recruitment according to the eligibility criteria, project explanation, detection of informed consent, assignment of a personal anonymous code, administration of the first (entrance) questionnaire, administration of a breath test to evaluate the blood alcohol concentration, and measurement of the driving reaction time. At the exit of the club, scheduled activities include the administration of the second (exit) questionnaire, alcohol test and driving reaction time measurement, along with a drug test for the first time. Then, the sheet with results (if requested), and the project gadget (an iPod holder) are given to the participants.

In each country, the survey staff is composed of at least six operators that have been specifically trained, including a survey manager (responsible for supervising all field activities), one technician for the reaction time evaluation, two interviewers (responsible for questionnaires and test administration) and two hostesses for approaching young people. Prior to conducting interventions, staff members from each country attended a two-day training session with the study coordinator.

### Confidentiality

Precautions are taken to guarantee data confidentiality. To maintain a rigorously anonymous management of the data while keeping the link between biological tests and survey responses for each respondent, a unique identifier is generated that specified the individual participant. The adopted method involves disposable, non-transferable bracelets [28] that possess a guaranteed adhesive closure and are waterproof and pre-printed with progressive identification numbers. At the entry interview, the respondent is provided with a bracelet possessing the unique identifying number and is asked not to remove it. People who do not agree to wear the bracelet are excluded from the study. The identification number is noted on the packet, including the questionnaires and the test results form. On exit from the venue, the id number on the bracelet of the participant is matched with the corresponding packet. The bracelet is removed from the participant upon completion of the exit assessment to avoid any future link between the person and the tests results. At no time are the participants requested to provide their names or any other personal identifying information.

### Ethical aspects

The study is conducted according to the European Commission Ethical Code [29]. The TEND by Night project was approved by the Internal Central Ethics Committee, made up of the senior investigators from all participating countries involved in the project "see Additional file 2", and by the Central Ethics Committee of the Catholic University of the Sacred Heart in Rome "see Additional file 3".

An information letter (containing contacts) and an informed consent to be signed are given to the participant at the time of enrolment. The informed consent sheet is written in a language as plain as possible [30], so that it is understandable to young people, and it has been translated into all partners' languages. There are no exceptions in asking for written agreement from all countries involved in the project field actions [31], except for Belgium and Netherlands, where it is explicitly forbidden by local privacy laws.

### Instruments

#### The questionnaires

The entrance and exit questionnaires were developed based on validated questionnaires [32-34], tested for comprehensiveness, and translated into the national language of the respective country. The anonymous entrance questionnaire, is composed of 27 closed- and open-ended questions and consists of five sections that investigate the following topics:

1) Demographic data and socioeconomic status (gender, age, scholarity, occupational status, living conditions);

2) Medications consumption (medication consumption in the last seven days);

3) Driving habits (licensing age, driving frequency, speeding tickets and license suspensions received, involvement in traffic crashes);

4) Alcohol and drug use (usual and current consumption for a single substance);

5) Driving and substance use (driving experiences under the effects of alcohol or drugs, passenger experience when the driver was under the effects of alcohol or drugs, opinions about drunk and drugged driving).

The entrance questionnaire is estimated to take five to ten minutes to be compiled.

The anonymous exit questionnaire, composed of nine closed- and open-ended questions, consists of three sections that investigate the following topics:

6) Alcohol and drug consumption during the event;

7) Intention to drive upon exit;

8) Opinion about the intervention.

The exit questionnaire is estimated to take less than five minutes to be compiled.

The full questionnaire is available at the project website [25].

#### Alcohol test

The Dräger Alcotest 6510 [35] was chosen to detect participants' breath alcohol concentration (BAC). This tool, provided with the alcohol-specific Electrochemical Dräger Sensor in 1/4" technology, is listed on the National Highway Traffic Safety Administration Conforming Products List (NHTSA CPL) as an Evidential Breath Tester for Department of Transportation applications. The measurement results are given in g/L, and their standard deviation is +- 1.7% of the measurement value. Reliable breath testing requires an alcohol-free mouth for 15 minutes preceding the administration of the test. The result is displayed after 10 seconds on average.

#### Drug test

We collect the saliva samples with the Oratect III Oral Fluid Drug Screen Device manufactured by the Branan Medical Corporation, which is a recent member of the devices available for that purpose [36]. The collector is a one-step chromatographic immunoassay device employed for the qualitative simultaneous detection of multiple drugs: cocaine, methamphetamine/MDMA, THC, amphetamine, opiates, benzodiazepines. The absence of a purple-red band at the test region indicates a presumptive positive result for that particular test. The test results are positive at or above the following cut-off drug concentrations: 25 ng/ml for d-methamphetamine/MDMA; 40 ng/ml for delta-9-tetrahydrocannabinol; 20 ng/ml for cocaine; 25 ng/ml for d-amphetamine; 10 ng/ml for morphine; 5 ng/ml for diazepam. The precision and specificity of the test are satisfactory for every substance considered [36]. The presence of the control band at the control region indicates that the test has performed properly. Saliva collection should last between 5 and 10 minutes, and the results should be read within 5 minutes after removing the device from mouth [36,37]. If the test is invalid, it is repeated.

#### Driving simulator

We measure the reaction time through a driving simulator: the SimuNomad3 Ecrans provided with the Software SCAM 03 and manufactured by the Ediser [38]. The driving simulator features a realistic functional dashboard, a virtual environment (with a field of view that was 90° horizontal by 45° vertical), three monitors and an ergonomic driver's seat. The system is fitted with realistic operational control instrumentation to facilitate normal braking, acceleration and cornering. Subjects accelerate, and a barrier fence suddenly appears at a random distance on the monitor in the direct path of the vehicle. Subjects are instructed to brake as quickly as possible to stop the vehicle and avoid hitting the fence. Reaction time is recorded as the latency time to release the gas pedal (gas-pedal-release latency), along with the latency time to press the brake pedal (brake latency), after the appearance of the fence.

During the first entrance test, the technician invites the participant to accelerate and drive along the road and to brake when obstacles appear on the roadway without steering the wheel. This test is only a demo for the participant to understand what to do. The driver is then invited to repeat the test: in this test, the reaction time value is noted. During the exit test, the driver performs the test only once.

### Pilot study

Prior to the main study, pilot studies are on course in every country involved. Approximately 250 young drivers of both sexes are being recruited to evaluate the feasibility of the project and to ensure the quality of procedures. Similarly, the validity and acceptability of study instruments is on evaluation.

### Statistical analysis

Initial descriptive statistics will be used to describe the sample: continuous variables will be expressed in terms of means and standard deviations if normally distributed (Shapiro-Wilk test), or medians and interquartile ranges; percentages will be used to describe categorical variables. All the results will be provided for the overall sample and stratified according to alcohol and the various drugs evaluated consumption (cocaine, methamphetamine/MDMA, THC, amphetamine, opiates, benzodiazepines).

The main outcome will be the difference in reaction time (RTD) between the entrance and exit test, and its correlation with alcohol and other drugs use, treating each consumption as a dichotomous variable (yes or not, given that it is not self-reported). For each psychoactive substance, the RTD of individuals consuming the substance will be compared with that of individuals not consuming the same substance, using Kruskal-Wallis test and two-way ANOVA. The potential confounding effect by other variables such as age or gender (or other psychoactive substances) will be accounted using multivariate regression.

As a secondary outcome it will be explored the relationship between reaction time difference and the amount of alcohol/drug consumption (as a continuous variable), separately for each psychoactive substance. After an initial evaluation using Spearman test, a linear regression model will also be developed. Indeed, such an outcome is of crucial importance, and it will be secondary only for drug consumptions, because their amount is only self-reported, whereas for alcohol consumption, the amount of which is more precisely estimated by the study sensor, it will be the main outcome.

Finally, the potential existence of threshold(s) for reaction time difference will also be explored attempting several cutoffs and testing their relationship with the amount of consumption of the various substances in univariate analysis. In case of promising cutoff values, binomial and/or ordinal multivariate logistic regression will be set to evaluate the independent predictors of each cutoff value of reaction time difference.

Standard procedures for model building and subsequent validity assessment will be adopted for both linear (transformation, if needed, of the dependent variable, stepwise forward based upon clinical relevance, univariate p < 0.10 and R^2 ^or >20% OR change, interaction - especially among various types of consumption - and higher power terms evaluation, multicollinearity and influential observation/residuals analysis - studentized, Cook's D influence, Hat diagonal matrix, Welsh distance, etc.) and logistic regression (in addition to the above - opportunely revised for logistic models - Hosmer-Lemeshow test for the goodness of fit, area under the Roc curve for the predictive power). In the linear regression for the secondary outcome only, the non-linear effect of covariates will be modeled using a restrictive cubic-spline function and its significance and will be assessed by the Wald's chi-square test. Interaction among variables will be checked using the same lines. Model fit will be considered as significantly improved for the latter on the basis of the Akaike Information Criterion (AIC) applied backwards for each model (i.e. starting from a model with all relevant variables, eliminating those that were not significant), at a significance level of 0.10.

A two-tailed p-value of 0.05 will be considered significant for all analyses, which will be carried out using STATA statistical software, version 10.0 (Stata Corp., College Station, TX, 2007).

## Discussion

The TEND by Night project is a large, multi-centric European survey that has been primarily designed to evaluate the association between alcohol and psychoactive substance consumption and driving ability in young people.

The project methodology should provide some relevant advantages over more traditional survey systems. On one side, most of the studies that have examined the patterns of consumption of alcohol and drugs in recreational places or in social settings used only self-reported measurements [39-42]. On the other side, some recent studies have evaluated the effective consumption of drugs and alcohol using biological measures at the entrance and exit of recreational sites, but they were performed only in very specific settings [27,43,44]. Our procedures combine self-reported alcohol and drugs use with the results of biological samples, minimizing the potential for recall bias. In addition, the portal survey technique performs a temporal evaluation of substance use and provides pre- and post-assessment of consumption, assuring the most reliable measure of substance effects.

As mentioned, two naturalistic studies have prospectively evaluated the potential relationship between alcohol and other psychoactive substances consumption with driving impairment indicators in recreational places [22,23]. These two studies, however, were focused on one psychoactive substance only and on relatively small samples. The TEND by Night is the first large-scale study on the topic and, to our knowledge, it is the first study in which a portal survey technique is applied both to several substances consumption and a driving performance indicator. The inclusion of the reaction time measurement allows the estimation of the co-occurrence of substances related harm and it substantiates the need for preventive interventions in high-risk settings to decrease the likelihood of co-occurring hazards such as driving under alcohol or drug influence.

The study has some limitations that must be mentioned. First, the voluntary recruitment of the participants and the opportunistic selection of the recreational venues could introduce an important selection bias, limiting the representativeness of the sample and the generalization of the final results on the prevalence of alcohol and drug consumption. However, such an issue does not impair in any way the main outcomes of the study, which is the relationship between consumption and driving ability. Second, the findings from a driving simulation do not perfectly correspond to on-road circumstances, and our results may not be perfectly applied towards on-road circumstances. However, in direct comparison studies, simulator results correlated well with real driving performances [45,46]. Finally, as regards the study instruments, while the measurement of alcohol allows the determination of an exact quantification, the measurement of drugs is exclusively dichotomic (presence/absence).

## Conclusions

Although the role of psychoactive substances in impairing driving ability is documented, the precise relationship between consumption and degree of driving impairment is not fully ascertained for several substances. The TEND by Night project has been designed to meet this challenge adopting an up-to-date methodology on a large sample of young individuals from recreational or social sites in five European countries.

## Competing interests

The authors declare that they have no competing interests.

## Authors' contributions

All the authors contributed to the conception, design and are currently carrying out the TEND by Night study. RS, SCP and LM wrote the present manuscript. All authors read and approved the final manuscript.

## Pre-publication history

The pre-publication history for this paper can be accessed here:

http://www.biomedcentral.com/1471-2458/10/205/prepub

## Supplementary Material

Additional file 1 Funding proof. Click here for file

Additional file 2 Internal Central Ethics Committee approval proof. Click here for file

Additional file 3 Central Ethics Committee of the Catholic University of the Sacred Heart in Rome approval proof. Click here for file
