# Supplementary material for: A European study on alcohol and drug use among young drivers: the TEND by Night study design and methodology
Source: BMC Public Health. 2010 Apr 26;10:205. doi: 10.1186/1471-2458-10-205 (PMC2873581; doi:10.1186/1471-2458-10-205)
Supplement: Additional file 1 — Funding proof. [file 1471-2458-10-205-S1.PDF]

GRANT AGREEMENT FOR AN ACTION WITH MULTIPLE BENEFICIARIES

AGREEMENT NUMBER – 2007326

The Public Health Executive Agency (PHEA) (hereinafter referred to as "the Executive Agency"), acting under powers delegated by the Commission of the European Communities ("the Commission"), and represented for the purposes of signature of this agreement by Mr. Luc Briol, Director, or his duly authorised representative,

of the one part,

and

CONSUSA-SERVIZI PIEMONTE SPA – **CONSEPI**
Private law Body
Official Registration Number: 578221/CF03719310017
Frazione Traduerivi, 12
I-10059 SUSA (TO)
ITALY
IVA Number: IT03719310017

hereinafter called "the co-ordinator", represented for the purposes of the signature of the present agreement by Mr. Claudio BONANSEA, Managing Director

and the following "co-beneficiaries":

- UNIVERSITA DEGLI STUDI DI TORINO – **UniTo** - established in Italy
- S. & T. SOC. COOP. A R.L. – **S&T** – established in Italy
- FUNDACJA KIEROWCA BEZPIECZNY – **Fundacja** – established in Poland
- SDRUZHENIE OTVORENA MLADEZH * OM OPEN YOUTH – **Open Youth** – established in Bulgaria
- FONDATION TANGUY MOREAU DE MELEN RESPONSIBLE YOUNG DRIVERS - SECURA FORUM – **RYD** – established in Belgium
- UNIVERSITAT DE VALENCIA ESTUDI GENERAL – **UVEG** – established in Spain
- RIGA STRADINA UNIVERSITATE * RIGA STRADINS UNIVERSITY – **RSU** – established in Latvia

who have conferred powers of attorney for the purposes of the signature of the present agreement to the representative of the co-ordinator,

collectively "the beneficiaries", and each individually identified as "beneficiary" for purposes of this agreement where a provision applies without distinction to the co-ordinator or a co-beneficiary

of the other part,

collectively *"the parties to the agreement"*

HAVE AGREED

the **Special Conditions, General Conditions** and **Annexes** below:

Annex I Description of the action [Technical Annex]

Annex II Estimated budget of the action [Financial Annex]

Annex III Reporting requirements

Annex IV Letters of mandates conferring powers of attorney from the co-beneficiaries to the co-ordinator]

which form an integral part of this agreement ("the agreement").

The terms set out in the Special Conditions shall take precedence over those in the other parts of the agreement.

The terms of the General Conditions shall take precedence over those in the Annexes.

I – SPECIAL CONDITIONS

ARTICLE I.1 – PURPOSE OF THE GRANT

I.1.1 The Executive Agency has decided to award a grant, under the terms and conditions set out in the Special Conditions, the General Conditions and the Annexes to the agreement, which the beneficiaries hereby declare that they have taken note of and accept, for the action entitled **TEND by Night – (Dark, Dance, Disco, Dose, Drugs, Drive, Danger, Damage, Disability, Death)** ("*the action*").

I.1.2 The beneficiaries accept the grant and undertake to do everything in their power to carry out the action as described in Annex I, acting on their own responsibility.

ARTICLE I.2 – DURATION

I.2.1 The agreement shall enter into force on the date when the last party signs.

I.2.2 The action shall run for **24 months** from **01.03.2008** (*"the starting date of the action"*).

ARTICLE I.3 – ROLE OF THE BENEFICIARIES

I.3.1 The co-ordinator shall 'inter alia':

a) have full responsibility for ensuring that the action is implemented in accordance with the agreement;

b) be the intermediary for all communication between the co-beneficiaries and the Executive Agency in accordance with Article I.8. Any claims that the Executive Agency might have in respect of the agreement shall be addressed to, and answered by, the co-ordinator, save where specifically stated otherwise in the agreement;

c) be responsible for supplying all documents and information to the Executive Agency which may be required under the agreement, in particular in relation to the requests for payment. The co-ordinator shall not delegate any part of this task to the co-beneficiaries or to any other party. Where information from the co-beneficiaries is required, the co-ordinator shall be responsible for obtaining and verifying this information and for passing it on to the Executive Agency;

d) inform the co-beneficiaries of any event of which the co-ordinator is aware that is liable to substantially affect the implementation of the action;

e) inform the Executive Agency of transfers between items of eligible costs, as provided in Article I.4.4;

PhP

f) make the appropriate arrangements for providing the financial guarantee or the joint guarantee of the beneficiaries participating in the action, when requested, under the provisions of Article I.5;

g) establish the payment requests on behalf of the beneficiaries, detailing the exact share and amount assigned to each beneficiary, in accordance with the agreement, the estimated eligible costs as foreseen in Annex II, and the actual costs incurred. All payments by the Executive Agency are made to the bank account(s) referred to in paragraph 1 of Article I.7;

h) where designated the sole recipient of payments on behalf of all of the beneficiaries, ensure that all the appropriate payments are made to the co-beneficiaries without unjustified delay in accordance with paragraph 3 of Article I.7 and shall inform the Executive Agency of the distribution of the Community financial contribution between the co-beneficiaries and of the date of transfer;

i) be responsible, in the event of audits, checks or evaluations, as described in Articles II.20 and II.6, for providing all the necessary documents, including the accounts of the co-beneficiaries, the original accounting documents and signed copies of sub-contracts, if any have been concluded by the beneficiaries in accordance with Article II.9.

I.3.2 The co-beneficiaries shall 'inter alia':

a) agree upon appropriate arrangements between themselves for the proper performance of the action;

b) forward to the co-ordinator the data needed to draw up the reports, financial statements and other documents provided for in the agreement including its Annexes;

c) ensure that all information to be provided to the Executive Agency is sent via the co-ordinator, save where the agreement specifically stipulates otherwise;

d) inform the co-ordinator immediately of any event liable to substantially affect or delay the implementation of the action of which they are aware;

e) inform the co-ordinator of transfers between items of eligible costs, as provided in Article I.4.4;

f) provide the co-ordinator with all the necessary documents in the event of audits, checks of evaluations, as described in Articles II.20 and II.6.

PM

ARTICLE I.4 – BREAKDOWN OF COSTS – FINANCING THE ACTION

I.4.1 The total cost of the action is estimated at **EUR 1.039.452,00 (one million and thirty nine thousand four hundred and fifty two euros)**, as shown in the estimated budget in Annex II. The estimated budget shall give a detailed breakdown of the costs that are eligible for Community funding under the terms of Article II.14, of any other costs that the action may entail, and of all receipts, so that receipts and costs balance.

The estimated budget in Annex II shall include a table indicating the breakdown of estimated eligible costs and receipts between each beneficiary. The table shall be agreed collectively by the beneficiaries and shall be deemed to form an integral part of the estimated budget of the agreement.

I.4.2 The total eligible costs of the action for which the Executive Agency grant is awarded are estimated at **EUR 1.039.452,00 (one million and thirty nine thousand four hundred and fifty two euros)**, as shown in the estimated budget in Annex II.

Indirect costs are eligible for flat-rate funding up to a maximum of 7 % of the total direct costs eligible, subject to the conditions laid down in Article II.14.3.

I.4.3 The Executive Agency shall contribute a maximum of **EUR 500.000,00 (five hundred thousand euros)**, equivalent to **48,10%** of the estimated total eligible costs indicated in Article I.4.2. The final amount of the grant shall be determined as specified in Article II.17, without prejudice to Article II.20.

The Community grant may not finance the entire costs of the action. The amounts and sources of co-financing other than from Community funds shall be set out in the estimated budget referred to in Article I.4.1.

I.4.4 By way of derogation from Article II.13, the co-ordinator may, in agreement with the co-beneficiaries, when carrying out the action, adjust the estimated budget by transfers between items of eligible costs, provided that this adjustment of expenditure does not affect the implementation of the action and the transfer between items does not exceed **20%** of the amount of each item of estimated eligible costs for which the transfer is intended, and without exceeding the total eligible costs indicated in Article I.4.2. The co-ordinator shall inform the Executive Agency in writing.

ARTICLE I.5 – PAYMENT ARRANGEMENTS

I.5.1 Pre-financing:

Within 45 days of the date when the last of the parties signs the agreement, a pre-financing payment of **EUR 150.000,00 (one hundred and fifty thousand euros)** shall be made to the co-ordinator, representing **30%** of the amount specified in Article I.4.3.

I.5.2 Further pre-financing payments:

Pre-financing may be paid in several instalments. In that case, payment of each further instalment to the co-ordinator may not be made until at least 30% of the previous pre-financing payment has been used up. Where the consumption of the previous pre-financing is less than 70%, the amount of the new pre-financing payment shall be reduced by the unused amounts of the previous pre-financing[1].

Every request for payment of a further pre-financing instalment must be accompanied by the documents specified in Article II.15.2.

Within 45 days after the Executive Agency receives and approves the request for payment of a further instalment, together with the documents referred to in the previous subparagraph, a pre-financing payment of **EUR 200.000,00 (two hundred thousand euros)** shall be made to the co-ordinator, equivalent to **40%** of the amount specified in Article I.4.3.

The period for payment referred to in the previous sub-paragraph of this article may be suspended by the Executive Agency in accordance with the procedure in Article II.16.2.

I.5.3 Payment of the balance:

The request for payment of the balance shall be accompanied by the documents specified in Article II.15.4.

The Executive Agency shall have 45 days to approve or reject the technical implementation report and the final financial statement or to request additional supporting documents or information under the procedure laid down in Article II.15.4. In that case, the co-ordinator shall have 20 days to submit the additional information or a new report.

A payment representing the balance of the grant determined in accordance with Article II.17 shall be made to the co-ordinator within 45 days following approval by the Executive Agency of the technical implementation report accompanying the request for payment of the balance. The Executive Agency may suspend the period for payment in accordance with the procedure in Article II.16.2.

ARTICLE I.6 – SUBMISSION OF REPORTS AND OTHER DOCUMENTS

The provisions relating to the submission of the technical implementation reports, financial statements and other documents referred to in Article I.5 are contained in Annex III.

The technical implementation reports, financial statements and other documents referred to in Article I.5 must be submitted by the co-ordinator in **2 copies** in **English** on the following dates:

---

[1] The new pre-financing instalment shall be reduced by the amount corresponding to the difference between the 70% threshold and the amount that was actually consumed. (Example: previous pre-financing 300 of which 100 (<70%) was consumed; calculation: 210 (70% threshold of 300) – 100 consumed = deduction of 110 from following pre-financing instalment).

PM

- Interim reports and other documents related to a request for a further pre-financing as specified in Article I.5.2. within 2 months following a period of **12 months** after the starting date of the action specified in Article I.2.2., covering the period **M1 to M12**;
- Final reports and other documents related to a request for payment of the balance as specified in Article I.5.3. within 2 months following the closing date of the action specified in Article I.2.2., covering the whole project duration.

ARTICLE I.7 – BANK ACCOUNT

I.7.1 All payments shall be made to the co-ordinator's bank account or sub-account denominated in euros, as indicated below:

Name of bank	**BANCA ANTONVENETA SPA**
Address of the branch	**60, CORSO G. FERRARIS - TORINO**
Precise denomination of the account holder	**CONSEPI SPA**
IBAN account code	**IT52P0504001000000000004165B**

I.7.2. This account or sub-account must identify the payments made by the Executive Agency for carrying out the action for which the grant is awarded. If the funds paid to this account yield interest or equivalent benefits under the law of the State on whose territory the account is opened, such interest or benefits shall, if they are generated by pre-financing payments, be recovered by the Executive Agency as specified in Article II.16.4.

I.7.3. Within 45 days of the day on which the bank account under I.7.1 has been credited, the co-ordinator shall transfer to each co-beneficiary the amounts corresponding to their participation in the action in accordance with their pro rata share of the estimated costs as defined in the breakdown in Annex II when pre-financing payments are made, and their share of validated costs actually incurred when other payments are made.

ARTICLE I.8 – GENERAL ADMINISTRATIVE PROVISIONS

I.8.1. Any communication in connection with the agreement shall be in writing, indicating the number of the agreement, the title and acronym of the action and shall be sent to the following addresses:

For the Executive Agency:

Technical reports, requests for payment and any other correspondence must be addressed to:

Public Health Executive Agency (PHEA)
Scientific Unit
HITEC 0/071

L-2920 Luxembourg
Phea@ec.europa.eu

Ordinary mail shall be considered to have been received by the Executive Agency on the date on which it is formally registered by the Executive Agency unit responsible referred to above.

For the co-ordinator:

Mr. Claudio BONANSEA, Managing Director
CONSUSA-SERVIZI PIEMONTE SPA
Frazione Traduerivi, 12
I-10059 SUSA (TO)

Telephone: +39 012232752
Fax: +39 0122623786
E-mail: guidasicura@consepi.it

I.8.2 Any communication from the Executive Agency to the co-ordinator and/or co-beneficiaries and vice versa shall be made via the co-ordinator, save where specifically indicated otherwise in the agreement.

ARTICLE I.9 – LAW APPLICABLE AND COMPETENT COURT

The grant is governed by the terms of the agreement, the Community rules applicable and, on a subsidiary basis, by the law of Luxembourg relating to grants.

The beneficiaries may bring legal proceedings regarding decisions by the Executive Agency concerning the application of the provisions of the agreement and the arrangements for implementing it before the Court of First Instance of the European Communities and, in the event of appeal, the Court of Justice of the European Communities.

ARTICLE I.10 – DATA PROTECTION

All personal data contained in the agreement, including its execution, or related to it shall be processed in accordance with Regulation (EC) No 45/2001 of the European Parliament and of the Council on the protection of individuals with regard to the processing of personal data by the Community institutions and bodies and on the free movement of such data. Such data shall be processed solely in connection with the implementation and follow-up of the agreement by the Executive Agency and the Commission, without prejudice to the possibility of passing the data to internal audit services, to the European Court of Auditors, to the Financial Irregularities Panel and/or to the European Anti-Fraud Office (OLAF) for the purposes of safeguarding the financial interests of the Community.

Beneficiaries may, on written request, gain access to their personal data and correct any information that is inaccurate or incomplete. They should address any questions regarding the processing of their personal data to the Executive Agency and the

Commission. Beneficiaries may lodge a complaint against the processing of their personal data with the European Data Protection Supervisor at any time.

ARTICLE I.11 – FURTHER SPECIFIC CONDITIONS

The following special conditions apply to this agreement:

I.11.1 The beneficiary shall submit the payment requests in accordance with article I.5, including the underlying financial statements, in euros. By way of derogation from Article II.16.1, any conversion of actual costs into euros shall be made by the beneficiary at the monthly accounting rate established by the Commission and published on its website for the first day of the month following the end of the reporting period[2].

I.11.2 Without prejudice to Article II.3.2, the beneficiaries grant the Executive Agency and the Commission the right to publish results and reports in hard copy or electronic form.

I.11.3 Without prejudice to Article II.5.1, unless the Executive Agency requests or agrees otherwise, all communications or publications by the beneficiaries collectively or one of the beneficiary individually, which are related to the action, including conferences, seminars, videos, electronic communications or printed matter shall include the following statement: "*This [insert appropriate description, e.g. publication, conference, etc.] arises from the project [insert project title] which has received funding from the European Union, in the framework of the Public Health Programme.*"

---

2 http://ec.europa.eu/budget/inforeuro/index.cfm?fuseaction=home&Language=en

II – GENERAL CONDITIONS

PART A – LEGAL AND ADMINISTRATIVE PROVISIONS

ARTICLE II.1 – LIABILITY

II.1.1 The beneficiaries shall be responsible for complying with any legal obligations incumbent on them.

II.1.2 The Executive Agency shall not, in any circumstances or on any grounds, be held liable in the event of a claim under the agreement relating to any damage caused during the action's execution. Consequently, the Executive Agency will not entertain any request for indemnity or reimbursement accompanying any such claim.

II.1.3 Except in cases of force majeure, the beneficiaries shall make good any damage sustained by the Executive Agency as a result of the execution or faulty execution of the action.

II.1.4 The beneficiaries shall bear sole liability vis-à-vis third parties, including for damage of any kind sustained by them while the action is being carried out.

ARTICLE II.2 – CONFLICT OF INTERESTS

The beneficiaries undertake to take all the necessary measures to prevent any risk of conflicts of interests which could affect the impartial and objective performance of the agreement. Such conflict of interests could arise in particular as a result of economic interest, political or national affinity, family or emotional reasons, or any other shared interest.

Any situation constituting or likely to lead to a conflict of interests during the performance of the agreement must be brought to the attention of the Executive Agency, in writing, without delay. The beneficiaries shall undertake to take whatever steps are necessary to rectify this situation at once. The Executive Agency reserves the right to check that the measures taken are appropriate and may demand that the beneficiaries take additional measures, if necessary, within a certain time.

ARTICLE II.3 – OWNERSHIP/USE OF THE RESULTS

II.3.1 Unless stipulated otherwise in the agreement, ownership of the results of the action, including industrial and intellectual property rights, and of the reports and other documents relating to it shall be vested in the beneficiaries.

II.3.2 Without prejudice to paragraph 1, the beneficiaries grant the Executive Agency the right to make free use of the results of the action as it deems fit, provided it does not thereby breach its confidentiality obligations or existing industrial and intellectual property rights.

ARTICLE II.4 – CONFIDENTIALITY

The Executive Agency and the beneficiaries undertake to preserve the confidentiality of any document, information or other material directly related to the subject of the agreement that is duly classed as confidential, if disclosure could cause prejudice to the other party. The parties shall remain bound by this obligation beyond the closing date of the action.

ARTICLE II.5 – PUBLICITY

II.5.1 Unless the Executive Agency requests otherwise, any communication or publication by the beneficiaries collectively or any one of the beneficiaries individually about the action, including at a conference or seminar, shall indicate that the action has received funding from the Community.

Any communication or publication by the beneficiaries collectively or any one of the beneficiaries individually, in any form and medium, shall indicate that sole responsibility lies with the author and that the Executive Agency is not responsible for any use that may be made of the information contained therein.

II.5.2 The beneficiaries authorise the Executive Agency to publish the following information in any form and medium, including via the Internet:

- the beneficiaries' names and addresses,
- the subject and purpose of the grant,
- the amount granted and the proportion of the action's total cost covered by the funding.

Upon a reasoned and duly substantiated request by the co-ordinator, the Executive Agency may agree to forgo such publicity if disclosure of the information indicated above would risk compromising the beneficiaries' security or prejudicing their commercial interests.

ARTICLE II.6 – EVALUATION

Whenever the Commission carries out an interim or final evaluation of the action's impact measured against the objectives of the Community programme concerned, the co-ordinator with the support of the co-beneficiaries undertake to make available to the Commission and/or persons authorised by it all such documents or information as will allow the evaluation to be successfully completed and to give them the rights of access specified in Article II.20.

ARTICLE II.7 – SUSPENSION

II.7.1 The co-ordinator, in agreement with the co-beneficiaries, may suspend implementation of the action if exceptional circumstances make this impossible or excessively difficult, notably in the event of force majeure. The co-ordinator shall inform the Executive Agency without delay, giving all the necessary reasons and details and the foreseeable date of resumption.

II.7.2 If the Executive Agency does not terminate the agreement under Article II.11.3, the beneficiaries shall resume implementation of the action as initially planned once circumstances allow and the co-ordinator shall inform the Executive Agency accordingly. The duration of the action might be extended by a period equivalent to the length of the suspension. In accordance with Article II.13, a supplementary written agreement shall be concluded to extend the duration of the action and to make any amendments that may be necessary to adapt the action to the new implementing conditions.

ARTICLE II.8 – FORCE MAJEURE

II.8.1 Force majeure shall mean any unforeseeable exceptional situation or event beyond the parties' control which prevents them from fulfilling any of their obligations under the agreement, was not attributable to error or negligence on their part, and proves insurmountable in spite of all due diligence. Defects in equipment or material or delays in making them available (unless due to force majeure), labour disputes, strikes or financial difficulties cannot be invoked as force majeure by the defaulting party.

II.8.2 A party faced with force majeure shall inform the other party without delay by registered letter with advice of delivery or equivalent, stating the nature, probable duration and foreseeable effects.

II.8.3 The party faced with force majeure shall not be held in breach of his obligations under the agreement if he's prevented from fulfilling them by force majeure. The parties shall make every effort to minimise any damage due to force majeure.

II.8.4 The action may be suspended in accordance with Article II.7.

ARTICLE II.9 – AWARD OF CONTRACTS

II.9.1 If the beneficiaries have to conclude contracts in order to carry out the action and they constitute costs of the action under an item of eligible direct costs in the estimated budget, they shall seek competitive tenders from potential contractors and award the contract to the bid offering best value for money; in doing so they shall observe the principles of transparency and equal treatment of potential contractors and shall take care to avoid any conflict of interests.

II.9.2 Contracts as referred to in paragraph 1 may be awarded only in the following cases:

a) they may only cover the execution of a limited part of the action;

b) recourse to the award of contracts must be justified having regard to the nature of the action and what is necessary for its implementation;

c) the tasks concerned must be set out in Annex I and the corresponding estimated costs must be set out in detail in the budget in Annex II;

d) any recourse to the award of contracts while the action is under way, if not provided for in the initial grant application, shall be subject to prior written authorisation by the Executive Agency;

e) the beneficiaries shall retain sole responsibility for carrying out the action and for compliance with the provisions of the agreement. The beneficiaries must undertake to make the necessary arrangements to ensure that the contractor waives all rights in respect of the Executive Agency under the agreement;

f) the beneficiaries must undertake to ensure that the conditions applicable to them under Articles II.1, II.2, II.3, II.4, II.5, II.6, II.10 and II.20 of the agreement are also applicable to the contractor.

ARTICLE II.10 – ASSIGNMENT

Claims for payments to be carried out by the Executive Agency may not be transferred.

In exceptional circumstances, where the situation warrants it, the Executive Agency may authorise the assignment to a third party of the agreement and payments flowing from it, following a written request to that effect, giving reasons, from the co-ordinator in agreement with the co-beneficiaries. If the Executive Agency agrees, it must make its agreement known in writing to the co-ordinator before the proposed assignment takes place. In the absence of the above authorisation, or in the event of failure to observe the terms thereof, the assignment shall not be enforceable against and shall have no effect on the Executive Agency.

In no circumstances shall such an assignment release the beneficiaries from their obligations to the Executive Agency.

ARTICLE II.11 – TERMINATION OF THE AGREEMENT

II.11.1 Termination by the co-ordinator

In duly justified cases, the co-ordinator, in agreement with the co-beneficiaries, may withdraw the beneficiaries' request for a grant and terminate the agreement at any time by giving 60 days' written notice stating the reasons, without being required to furnish any indemnity on this account.

If no reasons are given or if the Executive Agency does not accept the reasons, the agreement shall be deemed to have been terminated improperly, with the consequences set out in the fifth subparagraph of paragraph 5.

II.11.2 Termination of the participation of a beneficiary

In duly justified cases, the co-ordinator may request to terminate the participation of a beneficiary by giving 60 days written notice. The co-ordinator shall include with any such request to the Executive Agency the remaining beneficiaries' proposal to reallocate the tasks of that beneficiary or where relevant to nominate a replacement, the reasons for the termination of the participation and the opinion of the beneficiary whose participation is requested to be terminated.

In duly justified cases, any beneficiary may request the termination of his participation in the agreement. The request must be submitted to the Executive Agency by the co-ordinator by giving 60 days written notice stating the reasons.

If no reasons are given or if the Executive Agency does not accept the reasons, the participation shall be deemed to have been terminated improperly, with the consequences set out in the fifth subparagraph of paragraph 5.

The termination of the participation of the beneficiary concerned shall take effect on the date of the Executive Agency's approval. A written additional agreement shall be concluded to make any amendments necessary to adapt the action to the new implementing conditions resulting from the partial termination.

II.11.3 Termination by the Executive Agency

The Executive Agency may decide to terminate the agreement or the participation of any one or several beneficiaries participating in the action without any indemnity on its part, in the following circumstances:

a) in the event of a change to the beneficiary's legal, financial, technical, organisational or ownership situation that is liable to affect the agreement substantially or to call into question the decision to award the grant;

b) if a beneficiary fails to fulfil a substantial obligation incumbent on him under the terms of the agreement, including its annexes;

c) in the event of force majeure, notified in accordance with Article II.8, or if the action has been suspended as a result of exceptional circumstances, notified in accordance with Article II.7;

d) if a beneficiary is declared bankrupt, is being wound up or is the subject of any other similar proceedings;

e) if a beneficiary is found guilty of an offence involving his professional conduct by a judgment having the force of res judicata or if he is guilty of grave professional misconduct proven by any justified means;

f) if a beneficiary is guilty of misrepresentation or submits information or reports inconsistent with reality to obtain the grant provided for in the agreement;

g) if a beneficiary has intentionally or by negligence committed a substantial irregularity in performing the agreement or in the event of fraud, corruption or any other illegal activity on the part of a beneficiary to the detriment of the European Communities' financial interests. A substantial irregularity consists of any infringement of a provision of an agreement or regulation resulting

from an act or an omission on the part of a beneficiary which causes or might cause a loss to the Community budget.

II.11.4 Termination procedure

The procedure is initiated by registered letter, with advice of delivery or equivalent. The co-ordinator shall ensure that all beneficiaries are duly informed.

In the cases referred to in points (a), (b) and (d) of paragraph 3, the co-ordinator, in consultation with the co-beneficiaries, shall have 30 days to submit observations and take any measures necessary to ensure continued fulfilment of the beneficiaries' obligations under the agreement. If the Executive Agency fails to confirm acceptance of these observations by giving written approval within 30 days of receiving them, the procedure shall continue to run.

Where notice is given, termination shall take effect at the end of the period of notice, which shall start to run from the date when notification of the Executive Agency's decision to terminate the agreement or the participation of a beneficiary is received.

Where notice is not given in the cases referred to in points (c), (e), (f) and (g) of paragraph 3, termination shall take effect from the day following the date on which notification of the Executive Agency's decision to terminate the agreement or the participation of a beneficiary is received.

II.11.5 Effects of termination

In the event of termination of the agreement, payments by the Executive Agency shall be limited to the eligible costs actually incurred by the beneficiaries up to the date when termination takes effect, in accordance with Article II.17. Costs relating to current commitments that are not due to be executed until after termination shall not be taken into account.

The co-ordinator shall have 60 days from the date when termination of the agreement takes effect, as notified by the Executive Agency, to produce a request for final payment in accordance with Article II.15.4. If no request for final payment is received within this time limit, the Executive Agency shall not reimburse the expenditure incurred by the beneficiaries up to the date of termination and it shall recover any amount if its use is not substantiated by the technical implementation reports and financial statements approved by the Executive Agency.

Where termination affects the participation of a beneficiary, only those eligible costs actually incurred by the beneficiary concerned up to the date when termination of his participation takes effect, in accordance with Article II.17 shall be considered eligible. Costs relating to current commitments that were not due to be executed until after termination shall not be taken into account. The request for payment of the eligible costs incurred up to the date when the termination of the participation of the beneficiary concerned takes effect shall be included in the following payment request due according to the schedule laid down in Article I.6.

By way of exception, at the end of the period of notice referred to in paragraph 4, where the Executive Agency is terminating the agreement on the grounds that the co-ordinator has failed to produce the final technical implementation report and financial statement within the deadline stipulated in Article I.5 and the co-ordinator has still not complied

DNO

with this obligation within two months following the written reminder sent by the Executive Agency by registered letter with advice of delivery or equivalent, the Executive Agency shall not reimburse the expenditure incurred by the beneficiaries up to the date on which the action ended and it shall recover any amount if its use is not substantiated by the technical implementation reports and financial statements approved by the Executive Agency.

By way of exception, in the event of improper termination of the agreement by the co-ordinator, or a beneficiary's participation in the action, or termination by the Executive Agency on the grounds set out in points (e), (f) or (g) of paragraph 3, the Executive Agency may require the partial or total repayment of sums already paid under the agreement on the basis of technical implementation reports and financial statements approved by the Executive Agency, in proportion to the gravity of the failings in question and after allowing the co-ordinator, and where relevant co-beneficiaries concerned, to submit their observations.

ARTICLE II.12 – FINANCIAL PENALTIES

By virtue of the Financial Regulation applicable to the general budget of the European Communities, any one or several of the beneficiaries declared to be in grave breach of their obligations under the agreement shall be liable to financial penalties of between 2% and 10% of the value of their share of the grant in question, with due regard for the principle of proportionality.

This rate may be increased to between 4% and 20% in the event of a repeated breach in the five years following the first. The beneficiary concerned shall be notified in writing of any decision by the Executive Agency to apply such financial penalties.

ARTICLE II.13 – SUPPLEMENTARY AGREEMENTS

II.13.1 Any amendment to the grant conditions must be the subject of a written supplementary agreement. No oral agreement may bind the parties to this effect.

II.13.2 The supplementary agreement may not have the purpose or the effect of making changes to the agreement which might call into question the decision awarding the grant or result in unequal treatment of applicants.

II.13.3 Where the request for amendment is made by the co-ordinator, in agreement with the co-beneficiaries, he must send the request to the Executive Agency in good time before it is due to take effect and at all events one month before the closing date of the action, except in cases duly substantiated by the co-ordinator and accepted by the Executive Agency.

PART B – FINANCIAL PROVISIONS

ARTICLE II.14 – ELIGIBLE COSTS

II.14.1 To be considered as eligible costs of the action, costs must satisfy the following general criteria:

- they are incurred during the duration of the action as specified in Article I.2.2 of the agreement, with the exception of costs relating to final reports and certificates on the action's financial statements and underlying accounts;

- they are connected with the subject of the agreement and they must be indicated in the estimated budget annexed to it;

- they are necessary for the implementation of the action which is the subject of the grant;

- they are identifiable and verifiable, in particular being recorded in the accounting records of a beneficiary and determined according to the applicable accounting standards of the country where the beneficiary is established and according to the usual cost-accounting practices of the beneficiary;

- they comply with the requirements of applicable tax and social legislation;

- they are reasonable, justified, and comply with the requirements of sound financial management, in particular regarding economy and efficiency.

The beneficiaries' internal accounting and auditing procedures must permit direct reconciliation of the costs and revenue declared in respect of the action with the corresponding accounting statements and supporting documents.

II.14.2 The eligible direct costs for the action are those costs which, with due regard for the conditions of eligibility set out in Article II.14.1, are identifiable as specific costs directly linked to performance of the action and which can therefore be booked to it direct. In particular, the following direct costs are eligible provided that they satisfy the criteria set out in the previous paragraph:

- the cost of staff assigned to the action, comprising actual salaries plus social security charges and other statutory costs included in the remuneration, provided that this does not exceed the average rates corresponding to the beneficiary's usual policy on remuneration;

  The corresponding salary costs of personnel of national administrations are eligible to the extent that they relate to the cost of activities which the relevant public authority would not carry out if the project concerned were not undertaken;

DND

- travel and subsistence allowances for staff taking part in the action, provided that they are in line with the beneficiary's usual practices on travel costs or do not exceed the scales approved annually by the Commission;

- the purchase cost of equipment (new or second-hand), provided that it is written off in accordance with the tax and accounting rules applicable to the beneficiary and generally accepted for items of the same kind. Only the portion of the equipment's depreciation corresponding to the duration of the action and the rate of actual use for the purposes of the action may be taken into account by the Executive Agency, except where the nature and/or the context of its use justifies different treatment by the Executive Agency;

- costs of consumables and supplies, provided that they are identifiable and assigned to the action;

- costs entailed by other contracts awarded by a beneficiary for the purposes of carrying out the action, provided that the conditions laid down in Article II.9 are met;

- costs arising directly from requirements imposed by the agreement (dissemination of information, specific evaluation of the action, audits, translations, reproduction, etc.), including the costs of any financial services (especially the cost of financial guarantees). Such costs may also include specific costs incurred by the co-ordinator for fulfilling his responsibilities in his capability of the body responsible for the overall management of the action and the co-ordination of the beneficiaries.

II.14.3 The eligible indirect costs for the action are those costs which, with due regard for the conditions of eligibility described in Article II.14.1, are not identifiable as specific costs directly linked to performance of the action which can be booked to it direct, but which can be identified and justified by the co-ordinator or a co-beneficiary using their accounting system as having been incurred in connection with the eligible direct costs for the action. They may not include any eligible direct costs.

By way of derogation from Article II.14.1, the indirect costs incurred in carrying out the action may be eligible for flat-rate funding fixed at not more than 7% of the total eligible direct costs. If provision is made in Article I.4.2 for flat-rate funding in respect of indirect costs, they need not be supported by accounting documents.

II.14.4 The following costs shall not be considered eligible:

- return on capital;

- debt and debt service charges;

- provisions for losses or potential future liabilities;

- interest owed;

- doubtful debts;

- exchange losses;
- VAT, unless the beneficiary can show that he is unable to recover it according to the applicable national legislation;
- costs declared by a beneficiary and covered by another action or work programme receiving a Community grant;
- excessive or reckless expenditure;
- contributions in kind.

II.14.5 Not applicable.

II.14.6 By way of derogation from paragraph 3, indirect costs shall not be eligible under a grant for an action awarded to a beneficiary who already receives an operating grant from the Community budget during the period in question.

ARTICLE II.15 – REQUESTS FOR PAYMENT

Payments shall be made in accordance with Article I.5 of the Special Conditions.

II.15.1 – PRE-FINANCING

Pre-financing is intended to provide the beneficiaries with a float.

Where required by the provisions of Article I.5 on pre-financing, the co-ordinator shall furnish a financial guarantee from a bank or an approved financial institution established in one of the Member States of the European Union.

The guarantor shall stand as first call guarantor and shall not require the Executive Agency to have recourse against the principal debtor.

The financial guarantee shall remain in force until the final payment by the Executive Agency match the proportion of the total grant accounted for by pre-financing. The Executive Agency undertakes to release the guarantee within sixty (60) days following that date.

II.15.2 – FURTHER PRE-FINANCING PAYMENTS

Where pre-financing is divided into several instalments, the co-ordinator may request a further pre-financing payment once the percentage of the previous payment specified in the provisions of Article I.5 on further pre-financing has been used up. The request shall be accompanied by the following documents:

- a progress report on the technical implementation of the action;
- a detailed financial statement of the eligible costs actually incurred, including a consolidated statement and a breakdown between each beneficiary;
- where required by the above-mentioned provisions of Article I.5, a financial guarantee in accordance with paragraph 1;

DND

- any other documents in support of his request for further pre-financing.

The documents accompanying the request for payment shall be drawn up in accordance with the relevant provisions in Article I.6 and the annexes.

II.15.3 – Not applicable

II.15.4 – Payment of the balance

Payment of the balance, which may not be repeated, is made after the end of the action on the basis of the costs actually incurred by the beneficiaries in carrying out the action. It may take the form of a recovery order where the total amount of earlier payments is greater than the amount of the final grant determined in accordance with Article II.17.

By the appropriate deadline indicated in Article I.6, the co-ordinator shall submit a request for payment of the balance accompanied by the following documents:

- a final report on the technical implementation of the action;
- a final detailed financial statement of the eligible costs actually incurred, following the structure of the estimated budget, including a consolidated statement and a breakdown between each beneficiary;
- a full summary statement of the receipts and expenditure of the action including a consolidated statement and a breakdown between each beneficiary;
- any other documents in support of his request for payment of the balance.

The documents accompanying the request for payment shall be drawn up in accordance with the provisions of Article I.6 and the annexes.

If an external audit of the action's accounts is not required, the co-ordinator and the co-beneficiaries themselves shall certify that the information provided in their request for payment to the Executive Agency is full, reliable and true. They shall also certify that the costs incurred can be considered eligible in accordance with the agreement, that all receipts have been declared, and that the request for payment is substantiated by adequate supporting documents that can be checked.

On receipt of these documents, the Executive Agency shall have the period specified in Article I.5 in order to:

- approve the final report on the technical implementation of the action and the detailed financial statement;
- ask the co-ordinator for supporting documents or any additional information it deems necessary to allow the approval of the technical implementation report and the financial statement.;
- reject the documents referred to in Article I.5.3. and ask for the submission of additional information or a new report.

DW

Failing a written reply from the Executive Agency within the time limit for scrutiny indicated above, the report shall be deemed to have been approved. Approval of the report accompanying the request for payment shall not imply recognition of their regularity or of the authenticity, completeness and correctness of the declarations and information they contain.

If additional information or a new report is requested, the time limit for scrutiny shall be extended by the time it takes to obtain this information. The co-ordinator shall be informed of that request and the extension of the delay for scrutiny by means of a formal document. The co-ordinator shall have the period laid down in Article I.5 to submit the information or new documents requested.

Extension of the delay for approval of the report may delay the payment by the equivalent time.

Where a report is rejected and a new report requested, the approval procedure described in this article shall apply.

In the event of renewed rejection, the Executive Agency reserves the right to terminate the agreement by invoking Article II.11.3(b).

ARTICLE II.16 – GENERAL PROVISIONS ON PAYMENTS

II.16.1 Payments shall be made by the Executive Agency in euros. Any conversion of actual costs into euros shall be made at the monthly accounting rate established by the Commission and published on its website for the first day of the month following the end of the reporting period[3], unless the Special Conditions of the agreement lay down specific provisions.

Payments by the Executive Agency shall be deemed to be effected on the date when they are debited to the Executive Agency's account.

II.16.2 The Executive Agency may suspend the period for payment laid down in Article I.5 at any time by notifying the co-ordinator that his request for payment cannot be met, either because it does not comply with the provisions of the agreement, or because the appropriate supporting documents have not been produced or because there is a suspicion that some of the expenses in the financial statement are not eligible and additional checks are being conducted.

The Executive Agency may also suspend its payments at any time if a beneficiary is found or presumed to have infringed the provisions of the agreement, in particular in the wake of the audits and checks provided for in Article II.20.

The Executive Agency shall inform the co-ordinator as soon as possible of any such suspension by registered letter with advice of delivery or equivalent, setting out the reasons for suspension.

---

[3] http://ec.europa.eu/budget/inforeuro/index.cfm?fuseaction=home&Language=en

Suspension shall take effect on the date when notice is sent by the Executive Agency. The remaining payment period shall start to run again from the date when a properly constituted request for payment is registered, when the supporting documents requested are received, or at the end of the suspension period as notified by the Executive Agency.

II.16.3 On expiry of the period for payment specified in Article I.5, and without prejudice to paragraph 2 of this Article, the beneficiaries are entitled to interest on the late payment at the rate applied by the European Central Bank for its main refinancing operations in euros, plus three and a half points; the reference rate to which the increase applies shall be the rate in force on the first day of the month of the final date for payment, as published in the C series of the Official Journal of the European Union. This provision shall not apply to recipients of a grant which are public authorities of the Member States of the European Union.

Interest on late payment shall cover the period from the final date for payment, exclusive, up to the date of payment as defined in paragraph 1, inclusive. The interest shall not be treated as a receipt for the action for the purposes of determining the final grant within the meaning of Article II.17.4. The suspension of payment by the Executive Agency may not be considered as late payment.

By way of exception, when the interest calculated in accordance with the provisions of the first and second subparagraphs is lower than or equal to EUR 200, it shall be paid to the co-ordinator only upon demand submitted within two months of receiving late payment.

II.16.4 The Executive Agency shall deduct the interest yielded by pre-financing which exceeds EUR 50 000 as provided for in Article I.5 from the payment of the balance of the amount due to the beneficiaries. The interest shall not be treated as a receipt for the action within the meaning of Article II.17.4.

Where the pre-financing payments exceed EUR 750 000 per agreement at the end of each financial year, the interest shall be recovered for each reporting period. Taking account of the risks associated with the management environment and the nature of actions financed, the Executive Agency may recover the interest generated by pre-financing lower than EUR 750 000 at least once a year.

Where the interest yielded exceeds the balance of the amount due to the beneficiaries as indicated in Article II.15.4, or is generated by pre-financing referred to in the previous subparagraph, the Executive Agency shall recover it in accordance with Article II.19.

Interest yielded by pre-financing paid to Member States is not due to the Executive Agency.

II.16.5 The co-ordinator shall have two months from the date of notification by the Executive Agency of the final amount of the grant determining the amount of the payment of the balance or the recovery order pursuant to Article II.17, or failing that of the date on which the payment of the balance was received, to request information in writing on the determination of the final grant, giving reasons for any disagreement. After this time such requests will no longer be

DH

considered. The Executive Agency undertakes to reply in writing within two months following the date on which the request for information is received, giving reasons for its reply. This procedure is without prejudice to the beneficiaries' right to appeal against the Executive Agency's decision pursuant to Article I.9. Under the terms of Community legislation in this matter, such appeals must be lodged within two months following the notification of the decision to the applicant or, failing that, following the date on which the applicant learned of the decision.

ARTICLE II.17 – DETERMINING THE FINAL GRANT

II.17.1 Without prejudice to information obtained subsequently pursuant to Article II.20, the Executive Agency shall adopt the amount of the final payment to be granted to the beneficiaries on the basis of the documents referred to in Article II.15.4 which it has approved.

II.17.2 The total amount paid by the Executive Agency may not in any circumstances exceed the maximum amount of the grant laid down in Article I.4.3, even if the total actual costs eligible exceed the estimated total eligible costs specified in Article I.4.2.

II.17.3 If the actual eligible costs when the action ends are lower than the estimated eligible costs, the Executive Agency's contribution shall be limited to the amount obtained by applying the grant percentage of Community co-funding per beneficiary - specified in the table 'Budget by beneficiary' of Annex II, 'Estimated budget of the action' -, to the respective actual eligible costs approved by the Executive Agency per beneficiary.

II.17.4 The beneficiaries hereby agree that the grant shall be limited to the amount necessary to balance the action's receipts and expenditure and that it may not in any circumstances produce a profit for them.

Profit shall mean any surplus of total actual receipts attributable to the action over the total actual costs of the action. The actual receipts to be taken into account shall be those which have been established, generated or confirmed on the date on which the request for payment of the balance is drawn up by the co-ordinator for financing other than the Community grant, to which shall be added the amount of the grant determined by applying the principles laid down in paragraphs 2 and 3 of this article. For the purposes of this article, only actual costs falling within the categories set out in the estimated budget referred to in Article I.4.1 and contained in Annex II shall be taken into account; non-eligible costs shall always be covered by non-Community resources.

Any surplus determined in this way shall result in a corresponding reduction in the amount of the grant.

II.17.5 Without prejudice to the right to terminate the agreement under Article II.11, and without prejudice to the right of the Executive Agency to apply the penalties referred to in Article II.12, if the action is not implemented or is implemented poorly, partially or late, the Executive Agency may reduce the grant initially provided for in line with the actual implementation of the action on the terms laid down in the agreement.

II.17.6 On the basis of the amount of the final payment determined in this way and of the aggregate amount of the payments already made under the terms of the agreement, the Executive Agency shall set the amount of the payment of the balance as being the amount still owing to the beneficiaries. Where the aggregate amount of the payments already made exceeds the amount of the final grant, the Executive Agency shall issue a recovery order for the surplus.

ARTICLE II.18 – FINANCIAL JOINT RESPONSIBILITY

The beneficiaries agree to be irrevocably and unconditionally, jointly and severally responsible for any amount due to the Executive Agency by one of them which could not be honoured by the latter. The amount due to the Executive Agency will not exceed the maximum value of the contribution that could be granted to the beneficiaries in accordance with Article I.4.3, increased where applicable by interest on late payment.

The beneficiaries are not jointly responsible for financial penalties which could be imposed on any defaulting beneficiary in accordance with Article II.12.

ARTICLE II.19 – RECOVERY

II.19.1 Where an amount, paid by the Executive Agency to the co-ordinator in his capacity of recipient of all payments, is to be recovered under the terms of the agreement, the co-ordinator undertakes to repay the Executive Agency the sum in question, on whatever terms and by whatever date it may specify, even if he has not been the final recipient of the amount due. In the latter case, if payment has not been made by the due date, the Executive Agency reserves the right to recover directly the amount due from the final recipient.

Where such an amount to be recovered under the terms of the agreement was directly paid by the Executive Agency to a beneficiary, or if recovery is justified under Article II.12 of the agreement, the beneficiary concerned undertakes to pay the Executive Agency the sum in question, on whatever terms and by whatever date it may specify.

II.19.2 If the obligation to pay the amount due is not honoured by the date set by the Executive Agency, the amount due shall bear interest at the rate indicated in Article II.16.3. Interest on late payment shall cover the period between the date set for payment, exclusive, and the date when the Executive Agency receives full payment of the amount owed, inclusive.

Any partial payment shall first be entered against charges and interest on late payment and then against the principal.

II.19.3 If payment has not been made by the due date, sums owed to the Executive Agency may be recovered by offsetting them against any sums owed to the concerned beneficiary after informing him accordingly by registered letter with acknowledgment of receipt or equivalent, or, depending on the terms of the Special conditions, by calling in the financial guarantee provided in accordance with Article II.15.1. In exceptional circumstances, justified by the necessity to safeguard the financial interests of the Communities, the Executive Agency and/or the Commission may recover by offsetting before the due date of the payment. The beneficiary's prior consent shall not be required. If the recovery

remains unsuccessful under the provisions above, the Executive Agency shall hold all the beneficiaries collectively jointly responsible for the amount due in accordance with Article II.18.

II.19.4 Bank charges occasioned by the recovery of the sums owed to the Executive Agency shall be borne by the concerned beneficiary.

II.19.5 The beneficiaries understand that under Article 256 of the Treaty establishing the European Community, the Commission may adopt an enforceable decision formally establishing an amount as receivable from persons other than States. An action may be brought against such decision before the Court of First Instance of the European Communities.

ARTICLE II.20 – CHECKS AND AUDITS

II.20.1 The co-ordinator undertakes to provide any detailed information requested by the Executive Agency and/or the Commission or by any other outside body authorised by the Executive Agency and/or the Commission to check that the action and the provisions of the agreement are being properly implemented. Where the Executive Agency and/or the Commission so wishes, it may request such information to be provided directly by a co-beneficiary.

II.20.2 The beneficiaries shall keep at the Executive Agency's disposal all original documents, especially accounting and tax records, or, in exceptional and duly justified cases, certified copies of original documents relating to the agreement, stored on any appropriate medium that ensures their integrity in accordance with the applicable national legislation, for a period of five years from the date of payment of the balance specified in Article I.5.

II.20.3 The beneficiaries agree that the Executive Agency may have an audit of the use made of the grant carried out either directly by its own staff or by any other outside body authorised to do so on its behalf. Such audits may be carried out throughout the period of implementation of the agreement until the balance is paid and for a period of five years from the date of payment of the balance. Where appropriate, the audit findings may lead to recovery decisions by the Executive Agency and/or the Commission.

II.20.4 The beneficiaries undertake to allow Executive Agency staff and outside personnel authorised by the Executive Agency and/or the Commission the appropriate right of access to sites and premises where the action is carried out and to all the information, including information in electronic format, needed in order to conduct such audits.

II.20.5 By virtue of Council Regulation (Euratom, EC) No 2185/96 and Regulation (EC) No 1073/1999 of the European Parliament and the Council, the European Anti-Fraud Office (OLAF) may also carry out on-the-spot checks and inspections in accordance with the procedures laid down by Community law for the protection of the financial interests of the European Communities against fraud and other irregularities. Where appropriate, the inspection findings may lead to recovery decisions by the Executive Agency and/or the Commission.

II.20.6 The European Court of Auditors shall have the same rights as the Executive Agency and the Commission, notably right of access, as regards checks and audits.

DN

SIGNATURES

For the co-ordinator

Mr. Claudio BONANSEA
Managing Director

READ AND APPROVED

Signature CONSEPI S.p.A. L'Amministratore Delegato Claudio Bonansea

Done at SUSA,

Date 20-03-2008

For the Executive Agency

Mr. Luc BRIOL
Director

Signature

Done at Luxembourg,

Date 1/4/08

In duplicate in English
