# Supplementary material for: A European study on alcohol and drug use among young drivers: the TEND by Night study design and methodology
Source: BMC Public Health. 2010 Apr 26;10:205. doi: 10.1186/1471-2458-10-205 (PMC2873581; doi:10.1186/1471-2458-10-205)
Supplement: Additional file 2 — Internal Central Ethics Committee approval proof. [file 1471-2458-10-205-S2.PDF]

UNIVERSITÀ DEGLI STUDI DI TORINO
DIPARTIMENTO DI SANITÀ PUBBLICA E DI MICROBIOLOGIA

2007 326 - TEN D

The Ethical Committee of TEND by Night project, established as foreseen in the Annex 1 of the Agreement signed between Consepi S.p.a (on behalf of all project's partners) and the Public Health Executive Agency (acting under powers delegated by Commission of the European Communities) has approved the TEND by Night Study Protocol, after verification of its conformity to national regulations.

The Ethical Committee of TEND by Night project is composed by:

Roberta Siliquini – Italy (University of Turin)
Francisco Alonso – Spain (University of Valencia)
Axel Druart – Belgium and Netherland (Responsible Young Drivers)
Andrzej Kalitowicz – Poland (Safe Driver Foundation)
Lamberto Manzoli – Italy (University of Chieti)
Daniel Vankov – Bulgaria (Open Youth)
Anita Villerusa – Latvia (Riga Stradins University)

The President of the Ethical Committee

Prof. Roberta Siliquini

UNIVERSITA' DI TORINO
Scuola di Specializzazione
in Igiene e Medicina Preventiva
Via Santena, 5 bis - 10126 TORINO
Tel. 011.6705875 - Fax 011.670.5889
IL DIRETTORE
Prof.ssa ROBERTA SILIQUINI
