# Supplementary material for: A European study on alcohol and drug use among young drivers: the TEND by Night study design and methodology
Source: BMC Public Health. 2010 Apr 26;10:205. doi: 10.1186/1471-2458-10-205 (PMC2873581; doi:10.1186/1471-2458-10-205)
Supplement: Additional file 3 — Central Ethics Committee of the Catholic University of the Sacred Heart in Rome approval proof. [file 1471-2458-10-205-S3.PDF]

UNIVERSITÀ CATTOLICA DEL SACRO CUORE
FACOLTÀ DI MEDICINA E CHIRURGIA "AGOSTINO GEMELLI"

COMITATO ETICO

00168 Roma,

Prot.s.g.(A 241)/C.E./2010

COMITATO ETICO
Partenza
Protocollo P/221/CE/2010
Data 26/03/2010

Chiar.mo Prof. Lorenzo ORNAGHI
Magnifico Rettore

SEDE

e p.c. Gent.ma Prof.ssa Roberta SILIQUINI
Dipartimento di Sanità Pubblica e Microbiologia
Università degli Studi di Torino

Riunione del 22 Marzo 2010

Erano presenti alla riunione :

- Prof. Salvatore MANCUSO, *Componente "ex officio" Presidente f.f. Per delega del Magnifico Rettore*
- Prof. Roberto COPPOLA, Professore Ordinario di Chirurgia "Campus Biomedico"
- Prof Carlo PATRONO, Professore Ordinario - Direttore Istituto di Farmacologia del Policlinico Universitario
- Prof. Elio SALVAGGIO, Pediatra
- Prof. A.G. SPAGNOLO, Professore Ordinario, Direttore Istituto di Bioetica
- Dott.sa Laura VENTURA, Medico di Medicina Generale Territoriale
- Prof. Antonio VILLANI, Primario del Servizio di Anestesia e Rianimazione Ospedale "Bambino Gesù"
- Dott.sa Enrica Maria PROLI, Farmacista – Farmacia Interna
- Prof. Lamberto MANZOLI, Biostatistico - Università degli Studi "G. D'Annunzio" di Chieti-Pescara
- Prof. Vincenzo Lorenzo PASCALI, Professore Ordinario - Direttore dell'Istituto di Medicina Legale e delle Assicurazioni
- Prof. Carlo CALTAGIRONE, Direttore Scientifico – Fondazione S. Lucia IRCCS
- Prof. Giovanni SCHIAVONE Giurista – UCSC Milano

Componenti assenti:

- Dott. Andrea CAMBIERI, Direttore Sanitario del Policlinico Universitario "A. Gemelli"
- Dott. Roberto IADICICCO, Esperto operante nel settore dei mezzi di comunicazione di massa
- Prof. Mons. Sergio LANZA, Assistente Ecclesiastico Generale - Ordinario di Teologia Pastorale
- Sig.ra Rosalia GALLUZZO, Rappr. Ass. Volontari "Gemelli" (AVOG)
- Prof. Sac. Ferdinando CITTERIO, Rappresentante Istituto Toniolo
- Sig.ra Luciana MATTU, Caposala del Policlinico Universitario

Il Comitato Etico, riunito il 22 marzo u.s. per esprimere il proprio parere etico motivato sullo Studio presentato dalla Prof.ssa Roberta Siliquini, relativo alla ricerca **"TEN-D by night (dark, dance, disco, dose, drugs, drive, danger, damage, disability, death)"**,

ESAMINATA

la documentazione acclusa ed in particolare la scheda informativa ed il modulo di consenso informato, la sinossi (vers. **20 febbraio 2010**), la scheda tecnica del simulatore, il contratto, l'elenco centri, e la rimanente documentazione,

CONSTATATO CHE

il protocollo presentato:

- è giustificato scientificamente ed eticamente quanto al razionale, obiettivi;
- è giustificato quanto al disegno sperimentale;
- è giustificato quanto ai soggetti di sperimentazione;
- è giustificato quanto al rapporto rischi/benefici;
- è giustificato quanto alle informazioni fornite ai soggetti e alle modalità di richiesta del consenso;
- è giustificato in quanto alla numerosità campionaria e all'indagine statistica;
- fa riferimento ai codici deontologici (in particolare alla revisione corrente della dichiarazione di Helsinki e/o alle Norme di Buona Pratica Clinica (ICH-GCP) secondo l'all. 1 al D.M. 15.7.97) ed ai D.M. 18 e 19 marzo '98 e successive modificazioni ed integrazioni;
- tiene conto del Decreto Legislativo 24 giugno 2003, n.211: *attuazione della direttiva 2001/20/Ce relativa all'applicazione della buona pratica clinica nell'esecuzione delle sperimentazioni cliniche di medicinali per uso clinico;*
- è conforme alle disposizioni di legge ed alle conseguenti raccomandazioni del Comitato Etico dell'Università Cattolica in materia di rispetto della privacy;

ESPRIME PARERE FAVOREVOLE

Il Comitato Etico del Policlinico Gemelli, pur non essendo competente per la struttura che richiede l'approvazione, ma prendendo atto della impossibilità del Comitato Etico competente di esprimere un parere in quanto in fase di ricostituzione, decide di valutare lo studio in oggetto.
Dalla valutazione emerge che gli obiettivi sono coerenti con il razionale scientifico, il disegno appare pertinente e rilevante, l'informativa al paziente adeguata per i soli soggetti maggiorenni che verranno arruolati in Italia, ritiene che lo studio sia giustificato da un punto di vista etico-scientifico e pertanto lo approva all'unanimità.
Si precisa che il Comitato Etico non si esprime in termini di idoneità della struttura.

Si ricorda che la sperimentazione deve essere condotta con l'osservanza del Decreto Ministeriale 15.7.'97 che recepisce le Norme di Buona Pratica Clinica (ICH-GCP) e successive modificazioni. In particolare si sottolinea che dette Norme prevedono un adeguato risarcimento in caso di danno.

Si dichiara che questo Comitato è organizzato ed opera nel rispetto delle norme di buona pratica clinica (GCP-ICH) e degli adempimenti previsti dall'allegato I al Decreto Ministeriale 18.3.1998: "Linee Guida per l'Istituzione ed il funzionamento dei Comitati Etici".

Il Presidente f.f.del Comitato Etico
Prof. Salvatore Mancuso
